# Supplementary material for: Alpha-Synuclein Preformed Fibrils Induce Cellular Senescence in Parkinson’s Disease Models
Source: Cells. 2021 Jul 5;10(7):1694. doi: 10.3390/cells10071694 (PMC8304385; doi:10.3390/cells10071694)
Supplement: Supplementary file 1 [file cells-10-01694-s001.zip › cells-1274759-supplementary.pdf]

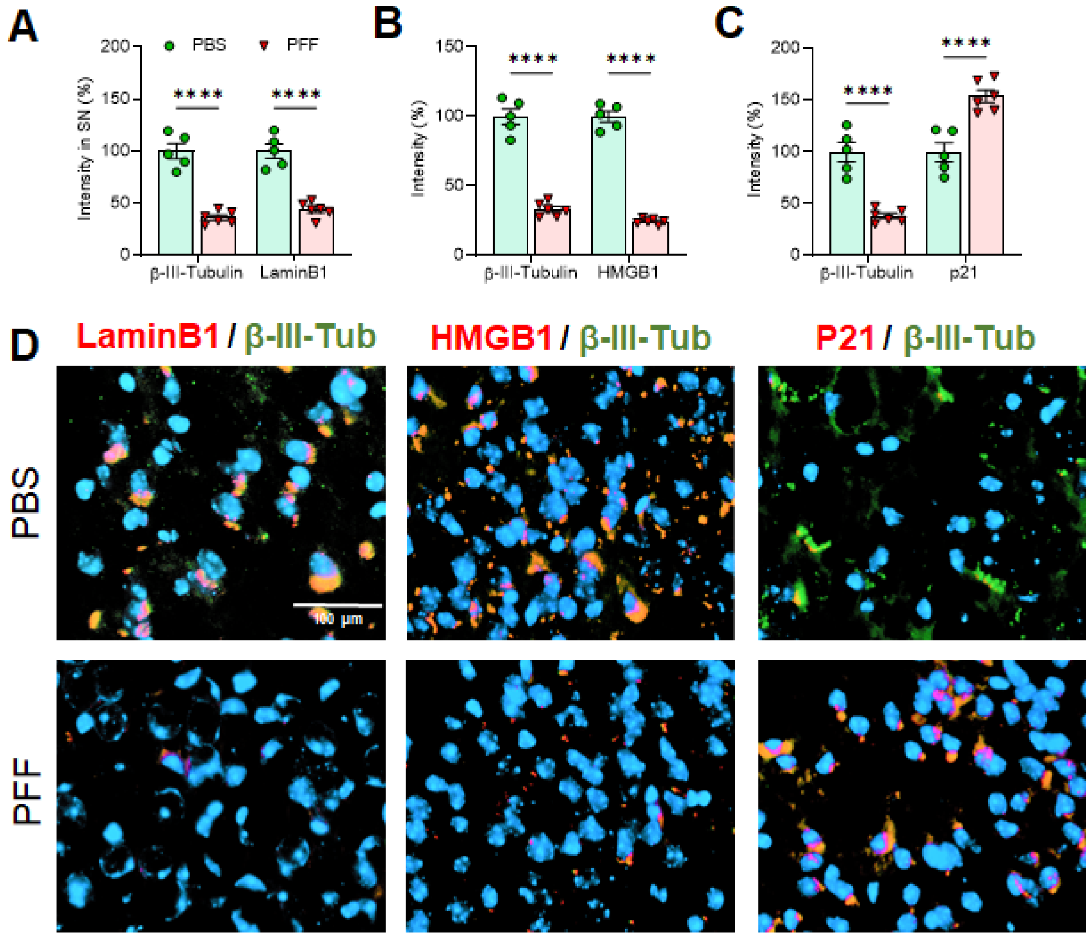

Figure S1. In the assessment of senescence markers in the SNc, the levels of LaminB1 and HMGB1 are lower in neurons in α-syn PFF-injected brains than PBS-treated ones, whereas the levels of p21 in neurons are increased with α-syn PFF. In the SNc, the label intensity of β-III-tubulin was decreased in PFF-injected SNc due to α-syn PFF injection (n=6/group). The intensities of LaminB1 and HMGB1 in neurons (**A**,**B**&**D**) were reduced, however, the levels of p21 were enhanced in neurons with α-syn PFF (**C**,**D**). DAPI stain (blue) was also used to indicate the location of nucleus. The label intensities of LaminB1, HMGB1 and p21 were quantified in β-III-tubulin-positive neurons, based on double-labels in a blinded manner. In quantification, PBS injected SNc region was used as a relative label intensity (100%, n=5/group) in mean±SEM and applied to unpaired Student's t-test for statistical significance. ****:p<0.0001. Size bar: 100μm.
